# Prevalence of social isolation before and during the COVID-19 pandemic: A nationwide web-based survey in Japan

**DOI:** 10.1093/geroni/igab046.2750

**Published:** 2021-12-17

**Authors:** Hiroshi Murayama, Ryo Okubo, Takahiro Tabuchi

**Affiliations:** 1 Tokyo Metropolitan Institute of Gerontology, Tokyo, Tokyo, Japan; 2 National Center of Neurology and Psychiatry, Kodaira, Tokyo, Japan; 3 Osaka International Cancer Institute, Osaka, Osaka, Japan

## Abstract

The coronavirus disease 2019 (COVID-19) pandemic is assumed to have increased the number of socially isolated older adults. Public health researchers and policymakers are concerned about the deleterious effects of social isolation on individuals’ health. However, there is only limited evidence on the prevalence of social isolation. This study investigated the change in prevalence of social isolation caused by the spread of COVID-19 and examined various associated factors. Accordingly, data from the JACSIS study, a nationwide cross-sectional web-based questionnaire survey (N=28,000, age: 15–79 years) conducted in August–September 2020 (during the pandemic) were analyzed. The respondents who contacted family members, friends, or neighbors less than once a week were considered socially isolated. We examined individuals’ frequencies of contact, including meeting in person, e-mail/text message, voice call, and video call, in January (before the pandemic; recall question) and August 2020. The weighted prevalence values of social isolation were 26.8% (26.0%–27.5%) in men and 15.8% (15.1%–16.4%) in women before the pandemic and increased to 34.4% (33.6%–35.2%) and 21.4% (20.7%–22.1%), respectively, during the pandemic. Further, compared to the younger age group, the increase in prevalence during the pandemic was greater for the older age group for both genders. Multinomial logistic regression analysis revealed that those who came to be socially isolated during the pandemic possessed a greater fear of COVID-19 than those who were not continuously socially isolated. These findings suggest the necessity of developing immediate measures for social isolation and risk communication regarding COVID-19.

